# Influence of harmonic waves on rectified diffusion around acoustic cavitation bubble under kHz ultrasound^☆^

**DOI:** 10.1016/j.ultsonch.2026.107995

**Published:** 2026-08-05

**Authors:** Kanji D. Hattori, Takuya Yamamoto

**Affiliations:** Department of Chemical Engineering, Graduate School of Engineering, Osaka Metropolitan University, 1-1, Gakuen-cho, Naka-ku, Sakai, Osaka 599-8531, Japan

**Keywords:** Keller-Miksis equation, Rectified diffusion, Harmonic wave, Diffusion stability

## Abstract

The influence of waveform deterioration on rectified diffusion around an acoustic cavitation bubble is numerically investigated by solving the Keller-Miksis equation and the advection–diffusion equation of dissolved gas around the bubble. The ultrasound waveform is constructed using a composite wave of fundamental and second harmonic waves. Numerical results indicated that, in cases involving harmonic waves, the maximum bubble radius during an ultrasonic oscillation is smaller due to instantaneous pressurization. This reduces the bubble growth due to rectified diffusion compared to case of linear waves. For harmonic waves with the phase shift in particular, the bubble expansion is largely suppressed, which reduces the bubble growth due to rectified diffusion. These results suggest that the reason why the power-induced quenching occurs when the ratio of harmonic waves is large and the number of formed bubbles is small, is caused by the decrease in the bubble growth due to smaller rectified diffusion. This is caused by the sound wave with harmonic waves and phase shift, which is generated by the sound emission from the oscillating bubbles.

## 1 Introduction

When a high-intensity ultrasound is irradiated into an aqueous solution, an acoustic cavitation occurs [1], [2]. At low-pressure zones created by an ultrasound, the local pressure becomes instantaneously much smaller than the vapor pressure, causing bubble nucleation. The nuclei are oscillated by the ultrasound repeatedly, and these bubbles grow due to rectified diffusion [2], [3], [4]. When the bubble grows sufficiently, the oscillation phase between the ultrasonic and bubble oscillations is shift, causing rapid collapse of bubble [5], [6]. At the moment of bubble collapse, the temperature and pressure in the bubble increases instantaneously and exceed 5000 K and 100 atmospheric pressure, respectively. In the created hot spot in the bubble, the molecules are thermally decomposed into smaller radicals, which cause many chemical reactions along the bubble interface and in the bubble [1]. When the bubble is collapsed, the shockwaves are emitted [7], and micro-jet forms especially under an asymmetric pressure conditions [8], [9]. In addition to the bubble dynamic motions, the acoustic streaming is developed in an ultrasonic bath due to the attenuation of ultrasound [10], [11]. Consequently, many physical and chemical phenomena occur simultaneously with different spatiotemporal scales. Although these phenomena are complicated to be understood, this technique has been utilized in many industrial applications including emulsification [12], [13], [14], [15], atomization [16], [17], particle dispersion [18], [19], [20], nanoparticles fabrications [21], [22], and organic chemical decomposition [23], [24], [25], [26]. The scope of this application has continued to expand.

The control of sonochemical reactions in an ultrasonic bath is difficult because many phenomena occur simultaneously as forementioned explanation. For example, the bubble clustering [27], [28], [29], [30], reflection plate [31], the particles in the liquid [32], [33], [34], the external flow [35], [36], and the concentration of dissolved gas [37], [38], [39] affect the rate of sonochemical reactions. The process is initiated by a phenomenon known as nucleation. However, rectified diffusion, which occurs subsequent to nucleation, contributes to the complexity of understanding and controlling the process. For example, the reaction zone and intensity are subject to variation depending on the dissolved gas concentration [40]. This phenomenon is thought to arise from alterations in the concentration of dissolved gas, which affect rectified diffusion, thereby influencing the bubble generation rate and the number density of bubbles. These changes, in turn, impact the reaction field. It is impossible to study each individual factors experimentally, because these phenomena are highly complex. We also pointed out that rectified diffusion is crucial when ultrasonic chemical reactions decrease [41], [42]. Under a high ultrasonic power density condition, the harmonics have amplitudes close to that of the fundamental frequency, causing significant distortion in the waveform. As clarified by our numerical study [43], these large distortion of ultrasonic wave is attributed to the sound emission and absorption from oscillations of acoustic cavitation bubbles. Under these conditions, the distorted ultrasonic wave reduces the magnitude of rectified diffusion and the chemical reaction rate. Rectified diffusion has been shown to play an instrumental role in sonochemical reactions; therefore, a thorough review of this subject is imperative.

The rectified diffusion has been investigated since Eller *et al*. numerically investigated [4], and summarized by Leighton [2]. Later, numerical simulation has been widely used to evaluate the rectified diffusion. For example, Louisnard *et al*. numerically investigated the rectified diffusion under the gas saturated condition [44], and Yoshizawa *et al*. [45] numerically solved the rectified diffusion under the ultrasound with two frequencies. So far, researchers investigated the rectified diffusion in molten metal [46], diffusive stability [47], effect of surface tension on the rectified diffusion [48], and the numerical model to calculate rectified diffusion has been improved [49]. Although, as forementioned explanation, the harmonics are generated by the acoustic emission from the bubbles and it affects the rectified diffusion, the quantitative evaluation has not been conducted so far especially under conditions of highly distorted ultrasound.

In this study, to shed light on the interaction between the large waveform distortion and rectified diffusion at excessive high-power ultrasound, we numerically investigated the rectified diffusion around an acoustic cavitation bubble under the composite waves of fundamental and second harmonic waves. We numerically investigated within a parameter range that observed in our previous experimental study particularly for the ratio of harmonic waves on the fundamental ultrasonic waves [41].

## 2 Numerical analysis

In this study, the rectified diffusion is numerically solved under an ultrasound with or without harmonic waves. The rectified diffusion is calculated by two equations: The Keller-Miksis equation and the advection–diffusion equation for the dissolved gas around an acoustic bubble. The Keller-Miksis equation is to calculate the oscillations of acoustic bubble under an ultrasound and the advection–diffusion equation is to calculate the mass transfer of dissolved gas around an acoustic bubble. By solving these two equations simultaneously, the rectified diffusion is capable of being calculated. In our experiment [41], we measured the time variation of dissolved oxygen concentration, and the degassing rate is strongly dependent on the ratio of harmonics. To model this phenomenon, we focused on the rectified diffusion of dissolved oxygen as a representative of dissolved gas.

### 2.1 The Keller-Miksis equation

The nonlinear oscillations of a spherical bubble were simulated using the following Keller-Miksis equation [1], [50] described as

(1) $$\begin{array}{c} \left(1-\frac{\dot{R}}{c_{\infty }}\right)R\ddot{R}+\frac{3}{2}{\dot{R}}^{2}\left(1-\frac{\dot{R}}{{3c}_{\infty }}\right)=\frac{1}{\rho_{l}}\left(1+\frac{\dot{R}}{c_{\infty }}\right)\left[p_{B}-p_{s}-p_{0}\right]+\frac{R}{c_{\infty }\rho_{l}}\frac{{dp}_{B}}{dt} \end{array}$$

where *R* is the bubble radius, $c_{\infty }$ is the sound velocity, $\rho_{l}$ is the liquid density, $p_{B}$ is the liquid pressure at the bubble wall, $p_{s}$ is the sound pressure, $p_{0}$ is the atmosphere pressure, and t is time.

The sound pressure with harmonic wave, *p*_s_ is given as

(2) $$\begin{array}{c} p_{s}=\alpha_{1}P\sin\left(-\omega_{1}t\right)+\alpha_{2}P\sin\left(-\omega_{2}t+\theta \right) \end{array}$$

where *P* is the pressure amplitude, $\omega_{1}$ and $\omega_{2}$ are the angular frequency of fundamental and second harmonic waves, θ is the phase shift, $\alpha_{1}$ and $\alpha_{2}$ are composition ratio of fundamental and second harmonic waves. To maintain the input ultrasonic power constant under different conditions, the following relationship is introduced [51].

(3) $$\begin{array}{c} \alpha_{1}^{2}+\alpha_{2}^{2}=1 \end{array}$$

The liquid pressure at the bubble wall, $p_{B}$ is expressed as

(4) $$\begin{array}{c} p_{B}=p_{g}+p_{v}-\frac{2\sigma }{R}-\frac{4\mu \dot{R}}{R} \end{array}$$

where $p_{g}$ is the pressure inside the bubble, $p_{v}$ is the vapor pressure, σ is the surface tension, and μ is the dynamic viscosity. The pressure inside the bubble, $p_{g}$ is described based on thermodynamic relationship as

(5) $$\begin{array}{c} p_{g}=\left(p_{0}+\frac{2\sigma }{R_{0}}-p_{v}\right){\left(\frac{R_{0}}{R}\right)}^{3\gamma } \end{array}$$

where γ is the ratio of specific heat.

In this numerical model, we limit the bubble wall velocity by sound speed in the liquid at the bubble wall [52], *c*_lB_, which is described as

(6) $$\begin{array}{c} c_{lB}=\sqrt{\frac{7.15\left(p_{B}+B\right)}{\rho_{lB}}} \end{array}$$

where *B* is the pressure parameter, $\rho_{lB}$ is liquid density at the bubble wall [1], [53].

### 2.2 Advection-diffusion equation of dissolved gas

The concentration distribution of dissolved gas around an oscillating bubble, *C*(*r*, *t*) is solved by the following advection–diffusion equation [47].

(7) $$\begin{array}{c} r^{2}\left(\frac{\partial C}{\partial t}\right)+R^{2}\dot{R}\left(\frac{\partial C}{\partial r}\right)=D\frac{\partial }{\partial r}\left(r^{2}\frac{\partial C}{\partial r}\right) \end{array}$$

where *r* is radial coordinate, and *D* is the diffusion coefficient. At the bubble wall boundary, the following Henry law is imposed.

(8) $$\begin{array}{c} C\left(R,t\right)=C_{0}\frac{p\left(R,t\right)}{p_{0}}=C_{0}\frac{p_{g}}{p_{0}} \end{array}$$

where *C*_0_ is the mass saturation concentration of dissolved gas. At infinity from the bubble wall, the following boundary condition is imposed.

(9) $$\begin{array}{c} C\left(\infty ,t\right)=C_{\infty } \end{array}$$

where $C_{\infty }$ is the bulk mass concentration of dissolved gas. In this simulation, the initial concentration was set to be uniform and the same as the bulk concentration. The bubble growth and dissolution rates can be calculated from the following mass change of bubble.

(10) $$\begin{array}{c} \dot{m}=4\pi R^{2}D{\left(\left(\frac{\partial C}{\partial r}\right)\right|}_{r=R\left(t\right)} \end{array}$$

The above numerical model is difficult to be solved numerically. Therefore, the following independent space variable is introduced [47].

(11) $$\begin{array}{c} r=F\left(x,t\right)={\left\{R^{3}\left(t\right)-R_{u}^{3}\ln\left(1-x\right)\right\}}^{\frac{1}{3}} \end{array}$$

where *R*_u_ is the adjustable length parameter, and *x* is the independent space variable. By using this independent space variable, Eq. (7) is finally transformed into

(12) $$\begin{array}{c} \frac{\partial (fC)}{\partial t}-\frac{\partial }{\partial x}\left(h\frac{\partial C}{\partial x}\right)=0 \end{array}$$

where *f* and *h* are described as

(13) $$\begin{array}{c} f\left(x,t\right)=\frac{R_{u}^{3}}{3(1-x)} \end{array}$$

(14) $$\begin{array}{c} h\left(x,t\right)=\frac{3D\left(1-x\right)F^{4}\left(x,t\right)}{R_{u}^{3}} \end{array}$$

In the same way as previous study [47], the maximum bubble radius is used for the adjustable length parameter. By using this procedure, the time-dependent independent space variable, $r\in \left[R\left(t\right),\infty \right]$ is mapped to the constant independent space variable, $x\in \left[0,1\right]$. Under this independent space variable, the boundary and initial conditions are transformed into

(15) $$\begin{array}{c} C\left(x=0,t\right)=C_{0}\frac{p_{g}}{p_{0}} \end{array}$$

(16) $$\begin{array}{c} C\left(x=1,t\right)=C_{\infty } \end{array}$$

(17) $$\begin{array}{c} C\left(x,0\right)=C_{\infty } \end{array}$$

The bubble growth and dissolution rates in this independent space variable are calculated as

(18) $$\begin{array}{c} \dot{m}=4\pi D\frac{R^{4}}{R_{u}^{3}}{\left(\left(\frac{\partial C}{\partial x}\right)\right|}_{x=0} \end{array}$$

The total mass change of bubble in an ultrasonic cycle, *M* is calculated as

(19) $$\begin{array}{c} M=\int_{0}^{T}\dot{m}dt \end{array}$$

where *T* is time of ultrasonic one cycle. In this study, the value of *M* is evaluated in a wide parameter range. The positive and negative values of *M* indicate the bubble growth and dissolution, respectively.

### 2.3 Other numerical models and parameters

The physical properties and parameters used in this study are shown in Table 1. We simulated the rectified diffusion under the experimental condition conducted in our previous study [41]. In this simulation, the equilibrium bubble radius and the pressure amplitude are widely changed because the ultrasonic pressure is spatially nonuniform in the bath. In this study, the equilibrium radius is varied from 0.60 to 1.80 μm with the interval of 0.025 μm, and the pressure amplitude is varied from 1.30 to 4.00 × 10^5^ Pa with the interval of 6.00 × 10^3^ Pa. The total number of numerical simulations to obtain one data is 48 × 45, i.e. 2160 runs. The fundamental frequency of ultrasound is 155 kHz, and second harmonics are generated due to the bubbles’ oscillations. As measured in our experiment [41], the ratio of fundamental wave is approximately 0.6–0.7 at least. Consequently, the ratio of fundamental wave used for this numerical simulation ranges from 0.7 to 1.0. Since the phase of the harmonics was shifted in the experiment, the phase shift is varied over a wide range.

**Table 1:** Physical properties and parameters used in this simulation.

Properties	Value	Unit
Liquid density, *ρ*_L,∞_	9.97 × 10^2^	kgm^−3^
External pressure, *p*_0_	1.00 × 10^5^	Pa
Vapor pressure, *p*_v_	3.166 × 10^3^	Pa
Liquid sound velocity, *c*_∞_	1.50 × 10^3^	ms^−1^
Liquid kinematic viscosity, *ν*	8.92 × 10^−7^	m^2^s^−1^
Surface tension, *σ*	7.20 × 10^−2^	Nm^−1^
Ratio of specific heat, *γ*	1.39	−
Saturation concentration of oxygen in water, *C*_0_	8.59 × 10^−3^	kgm^−3^
Bulk concentration of oxygen in water, *C*_∞_	8.59 × 10^−3^	kgm^−3^
Diffusion constant of oxygen, *D*	2.1 × 10^−9^	m^2^s^−1^
Pressure parameter, *B*	3.05 × 10^8^	Pa
Sound pressure amplitude, *P*	1.30–4.00 × 10^5^	Pa
Equilibrium bubble radius, *R*_0_	0.60–1.80 × 10^−6^	m
Fundamental angular frequency, *ω*_1_	155,000 × 2π	rads^−1^
Second harmonic angular frequency, *ω*_2_	310,000 × 2π	rads^−1^
Phase shift, *θ*	−π − π/2	rads^−1^
Ratio of fundamental wave, *α*_1_	0.7–1.0	−

The numerical code was self-developed in C++. The Keller-Miksis equation was discretized by 4th order Runge-Kutta method. The time increment of discretized equation was set to be 1.0 × 10^−3^ ns, which is sufficiently small to be independent of the time increment. The advection–diffusion equation for dissolved gas was discretized by the finite volume method. The spatial discretization scheme was second order central difference, and the temporal discretization scheme was first order explicit Euler method. The numerical grid is nonuniform and structured. The coordinate of numerical grid is determined by geometric sequence, the common ratio of which is set to be 1.05. The number of numerical grid points is changed depending on the pressure amplitude because the bubble oscillation amplitude is largely different according to the pressure amplitude. The number of numerical grid points is set to be 99 for *P* = 1.3 − 2.2 × 10^5^ Pa, and 155 for *P* = 2.2 − 4.0 × 10^5^ Pa except for *θ* = −π/2. In the case of *θ* = −π/2, the number of numerical grids is set to be 99 for all condition because the oscillation amplitude of bubble is not large.

The validation of our numerical code related to the Keller-Miksis equation has been conducted in our previous papers [54], [55]. Therefore, the validation results of the present simulation program are not shown in this study. In addition, the validation of numerical model particularly for the rectified diffusion was also performed by comparing the numerical results with those in the previous study [47]. The concentration distribution around the bubble as shown in Fig. 11 in Ref. [47] and the bifurcation diagram in the equilibrium radius – pressure amplitude parameter space as shown in Fig. 9 in Ref. [47] are predicted well. From above results, the developed program was determined to be capable of calculating rectified diffusion with sufficient precision.

## 3 Numerical results

### 3.1 Bubble growth and dissolution without phase shift

**Fig. 1:**
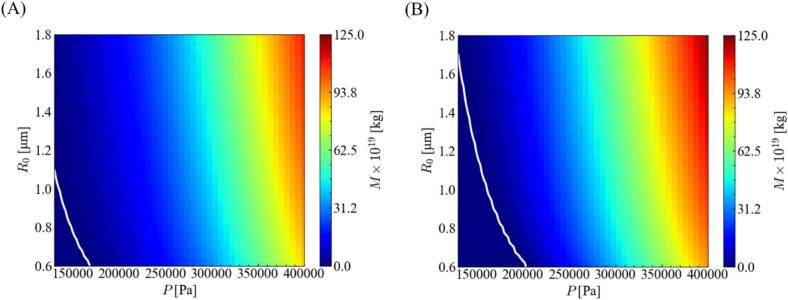
Distribution of the total mass change of the bubble in an ultrasonic cycle with and without harmonic waves; *α*_1_ = (A) 0.7 and (B) 1.0. The white line is the contour of *M* = 0.

Fig. 1 shows the distribution of total mass change of the bubble in an ultrasonic cycle with and without harmonic waves. In this figure, the white line is the contour of *M* = 0. The region on the left from the white line indicates the total mass change of the bubble becomes negative, which indicates the condition where the gas in the bubble dissolves into the liquid and the bubble shrinks. As shown in this figure, the white line moves toward left as the ratio of fundamental wave decreases. It means that the generation of harmonic waves suppresses the bubble shrinkage. Meanwhile, the maximum value of *M* becomes smaller as the ratio of fundamental wave decreases. It means that the generation of harmonic waves decreases the bubble growth rate at high pressure amplitude.

**Fig. 2:**
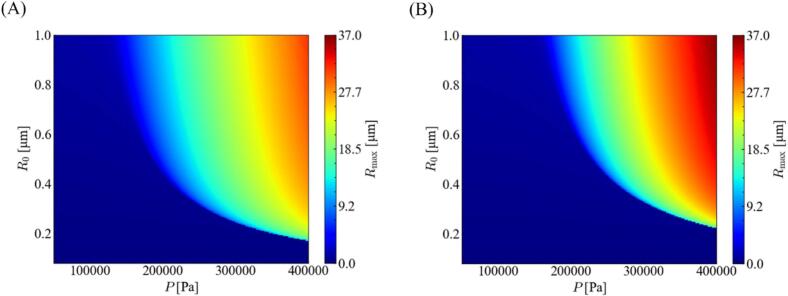
Distribution of the maximum bubble radius with and without harmonic waves; *α*_1_ = (A) 0.7 and (B) 1.0.

To understand the total mass change of the bubble in an ultrasonic cycle, we focus on the maximum bubble radius in an ultrasonic cycle. Fig. 2 shows the distribution of the maximum bubble radius with and without harmonic waves. The distribution shown in Fig. 1 is similar to that of Fig. 2. The rectified diffusion is mainly determined by *area effect* and *shell effect* [2]. The acoustic cavitation bubble expands and shrinks repeatedly. When the bubble expands, the inner pressure becomes small, causing inward mass flux of dissolved gas to satisfy the Henry law. At this time, the interfacial area becomes large, causing large inward mass flux, and this phenomenon is called *area effect*. On the other hand, when the bubble expands, the concentration boundary layer of dissolved gas along the bubble wall becomes thin, causing large inward mass flux. This phenomenon is called *shell effect*. In this result, the distribution of bubble radius is well corresponding to the distribution of total mass change of the bubble, suggesting that the *area effect* is dominant to determine the rectified diffusion within the investigated parameter range. The details about this dominant factor will be discussed later.

**Fig. 3:**
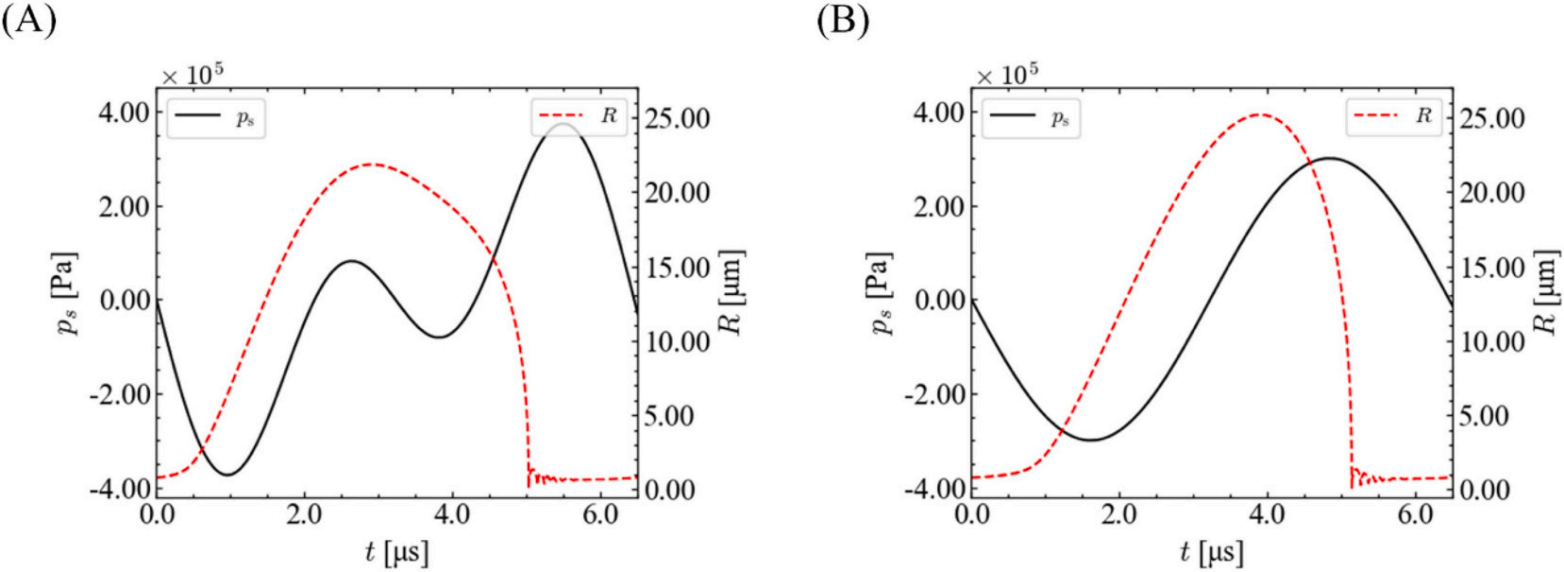
Time variations of the ultrasonic pressure and bubble radius for *P* = 3.0 × 10^5^ Pa, *R*_0_ = 0.8 µm with and without harmonic waves; *α*_1_ = (A) 0.7 and (B) 1.0.

Next, to understand the difference of the maximum bubble radius with different ratio of harmonic waves, we focused on the time variations of ultrasonic pressure and bubble radius. Fig. 3 shows the time variations of the ultrasonic pressure and bubble radius with and without harmonic waves. As an example, we selected the condition where *P* = 3.0 × 10^5^ Pa and *R*_0_ = 0.8 µm. When the harmonic wave is introduced, the waveform of imposed ultrasound is not sinusoidal. When the ultrasonic pressure decreases, the bubble expands and the bubble expansion continues due to inertia of liquid soon after the ultrasonic pressure starts to increase. In the case with harmonic wave, the bubble expansion is suppressed due to instantaneous pressurization by the harmonic wave, and finally the maximum bubble radius becomes small. This phenomenon is not limited to this condition, and it is consistent across the entire map particularly for the high-pressure condition.

**Fig. 4:**
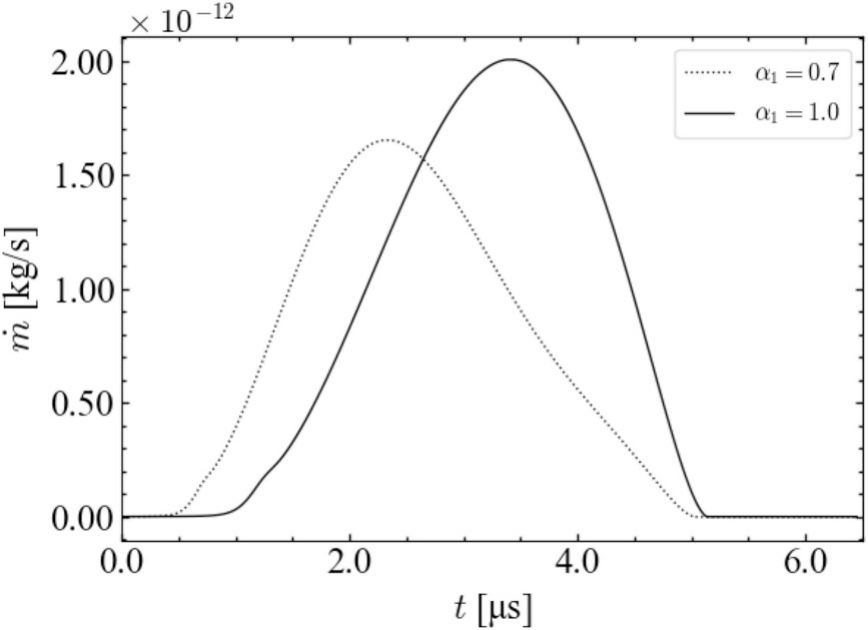
Time variation of the mass change rate of the bubble due to rectified diffusion with different ratios of fundamental frequency for *θ* = 0, *P* = 3.0 × 10^5^ Pa and *R*_0_ = 0.8 µm.

**Fig. 5:**
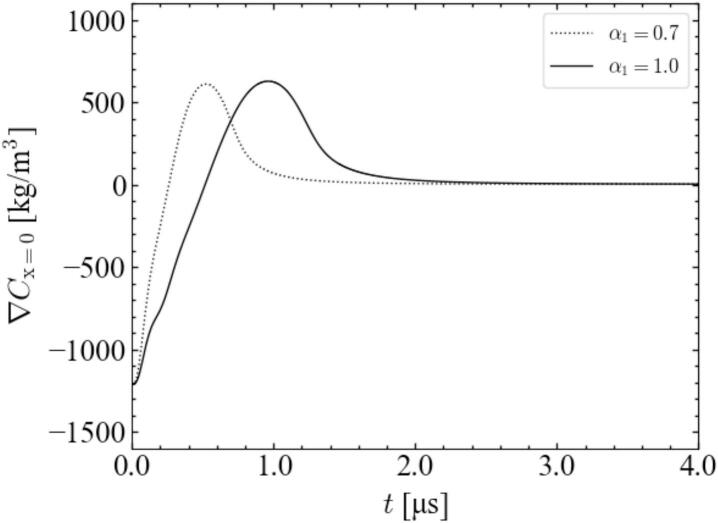
Time variation of the concentration gradient at the bubble wall during bubble expansion phase with different ratios of fundamental frequency for *θ* = 0, *P* = 3.0 × 10^5^ Pa and *R*_0_ = 0.8 µm.

To understand the details of rectified diffusion, the temporal change of mass change rate of the bubble and concentration gradient along the bubble wall are discussed. Fig. 4 shows the time variation of the mass change rate of the bubble due to rectified diffusion with different ratios of fundamental frequency, and Fig. 5 shows time variation of the concentration gradient at the bubble wall during bubble expansion phase with different ratios of fundamental frequency. The positive value in Fig. 4 means the bubble growth. The time at which the bubble radius reaches its maximum coincides with the time at which the mass change rate of the bubble reaches its maximum, although it does not coincide with the time at which the concentration gradient at the bubble wall reaches its maximum. From these results, it is considered that the main factor to determine the total mass change of the bubble is the interfacial area, and *area effect* is the dominant factor.

### 3.2 Bubble growth and dissolution with phase shift

**Fig. 6:**
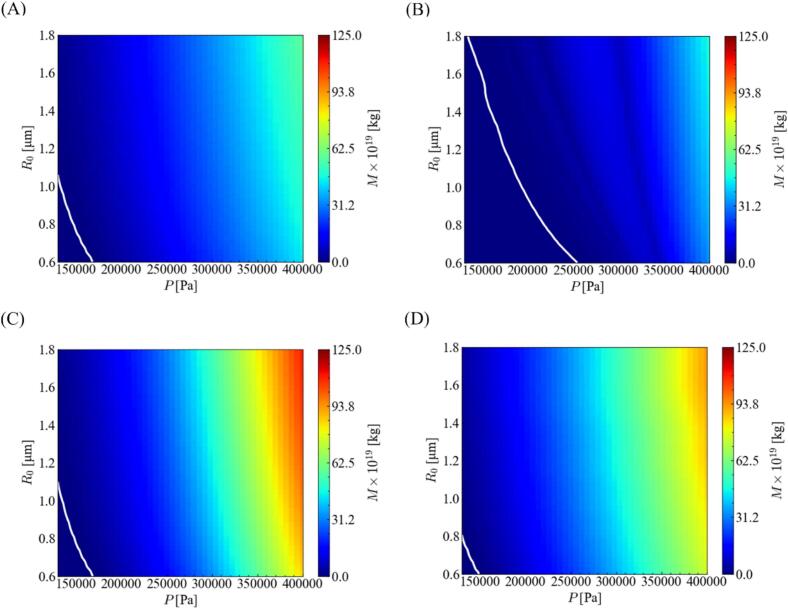
Distribution of the total mass change of the bubble in an ultrasonic cycle for the case of *α*_1_ = 0.7 with different phase shifts: *θ* = (A)-*π*, (B)-*π*/2, (C) 0, and (D) *π*/2. The region on the left from the white line indicates the total mass change of the bubble becomes negative, which indicates the condition where the gas in the bubble dissolves into the liquid and the bubble shrinks.

Next, the influence of phase shift on the total mass change of the bubble is discussed. Fig. 6 shows the distribution of the total mass change of the bubble in an ultrasonic cycle for the case of *α*_1_ = 0.7 with different phase shifts. In the same way as Fig. 1, the white line is the contour of *M* = 0. As can be seen clearly, the growth rate of the bubble decreases with increase in the magnitude of phase shift. Moreover, the growth rate is much smaller in the case with harmonic waves and phase shift than that without harmonic wave. Particularly for *θ* = −π/2, the growth rate of the bubble decrease largely and the shrinkage zone, that corresponds to the left zone from the white line, expands largely. In this case, the cavitation bubbles are difficult to be generated stably. The reason why the growth rate becomes small is discussed by comparing the numerical results without harmonic waves, i.e. α_1_ = 1.0 to those with harmonic waves and phase shift, i.e. α_1_ = 0.7 and *θ* = −π/2.

**Fig. 7:**
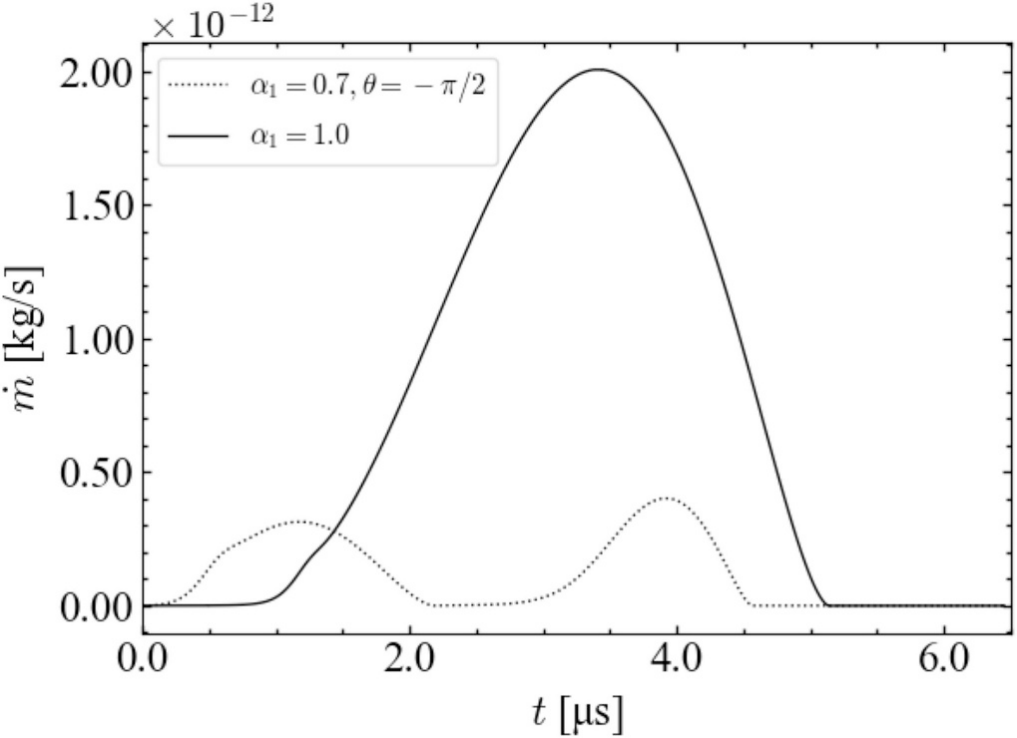
Time variation of the mass change rate of the bubble due to rectified diffusion with (*α*_1_ = 0.7 and *θ* = -*π*/2) and without harmonic waves (*α*_1_ = 1.0) for *P* = 3.0 × 10^5^ Pa and *R*_0_ = 0.8 µm.

**Fig. 8:**
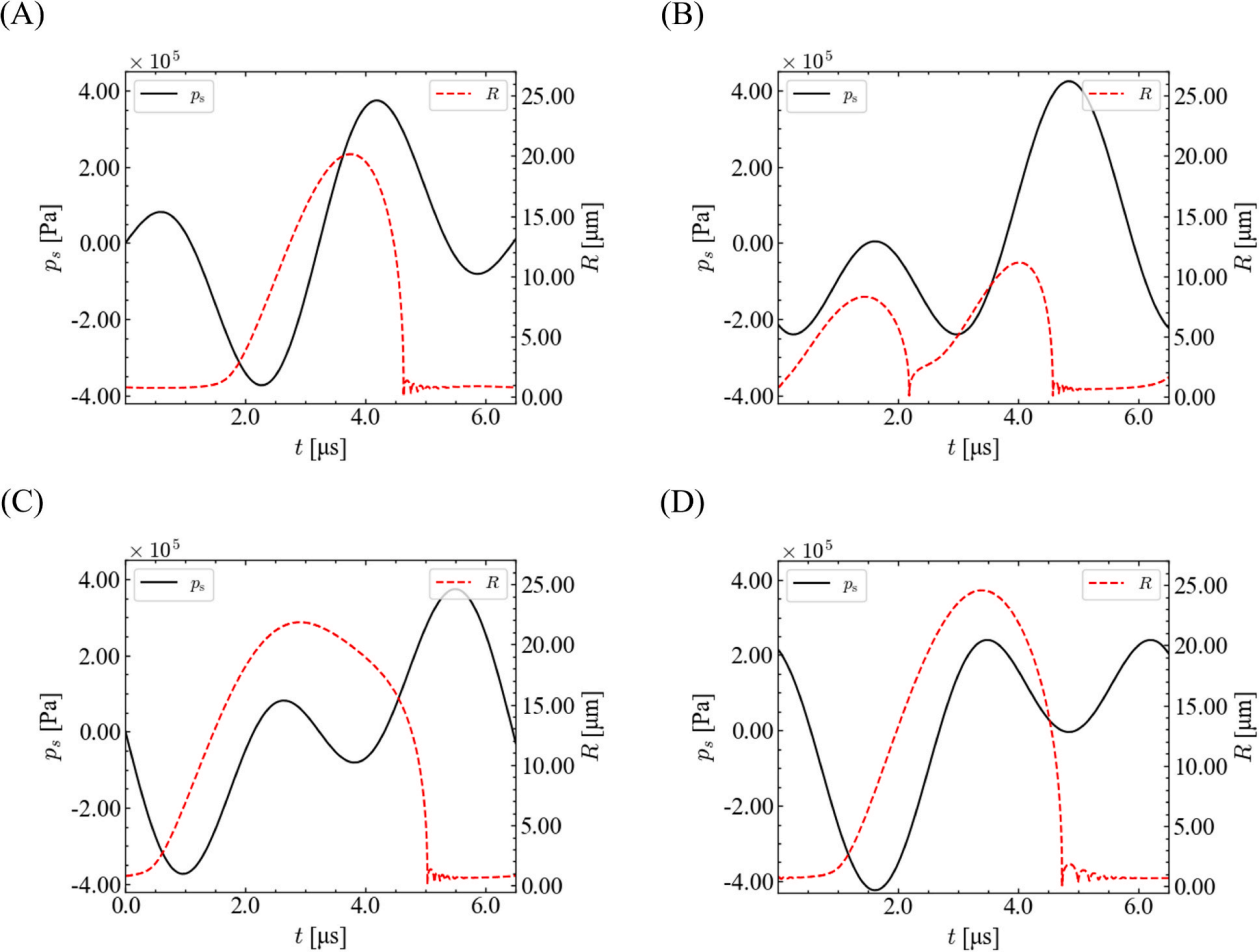
Time variations of the ultrasonic pressure and bubble radius for *P* = 3.0 × 10^5^ Pa, *R*_0_ = 0.8 µm and *α*_1_ = 0.7 with different phase shifts: *θ* = (A)-*π*, (B)-*π*/2, (C) 0, and (D) *π*/2.

Fig. 7 shows time variation of the mass change rate of the bubble due to rectified diffusion with and without harmonic waves. As an example, the mass change rate is shown for *P* = 3.0 × 10^5^ Pa and *R*_0_ = 0.8 µm. The growth rate increases largely in the case without harmonic wave, although it increases slightly twice in an ultrasonic cycle in the case with harmonic waves and phase shift. To understand the phenomena, the time variation of imposed ultrasound and bubble radius is focused. Fig. 8 shows time variations of the ultrasonic pressure and bubble radius with different phase shifts. The bubble rapidly shrinks twice in an ultrasonic cycle particularly for *P* = 3.0 × 10^5^ Pa, *R*_0_ = 0.8 µm and *θ* = −π/2. In accordance with the harmonic wave, the bubble is oscillated at twice the fundamental frequency of imposed ultrasound. In this case, the time for bubble expansion becomes extremely small, decreasing the maximum bubble radius. As discussed in section 3.1, the *area effect* governs the rectified diffusion in the investigated parameter range. Therefore, the bubble oscillation pattern with small bubble expansion is the main factor to reduce the bubble growth for *θ* = −π/2. Even when the phase shift differs from *θ* = −π/2, as shown in Fig. 8, the bubble is instantaneously pressurized, thereby suppressing bubble expansion compared with that for *θ* = 0. That is why the harmonic wave with phase shift largely reduces the growth rate due to rectified diffusion.

### 3.3 Discussion on the experimental results related to power-induced quenching

First, a comparison is made between the experimental results and the numerical results presented in this study. In circumstances where waveform distortion of ultrasound increases, the number of generated bubbles decreases [41]. In consideration of the results presented in Fig. 6, it is considered that the incorporation of harmonics with large phase differences into the waveform results in a substantial reduction in the rate of bubble growth. When ultrasound containing harmonics with large phase differences is irradiated to acoustic cavitation bubble, the bubble diameter becomes smaller. Smaller shrank cavitation bubbles after several ultrasonic cycles are more prone to dissolution as shown in Fig. 6. Consequently, once dissolution begins, it does not stop until the bubbles are completely dissolved. As a result, it is considered that the number of bubbles decreases.

Based on the above results, the possible mechanism of power-induced quenching [31], [41], [42], [43] is discussed. In our previous study [41], [42], the power-induced quenching occurs under the condition where the waveform of ultrasound is largely changed and the ratio of harmonic waves becomes large. Additionally, the number of formed bubbles decreases largely at the quenching condition [31], [41], [42]. Therefore, we considered that the quenching occurs in the following processes.

1. Harmonic waves with phase shift are generated due to the acoustic emission from the oscillating bubbles and wave superposition of the fundamental and emitted waves from the bubbles [43].
2. The oscillation pattern of cavitation bubbles is largely changed as shown in Fig. 8, and the bubble expansion is largely suppressed.
3. The bubble growth due to rectified diffusion is suppressed due to the oscillation pattern of bubble.
4. The degassing rate decreases accordingly after the quenching condition [41].

From above discussion, the harmonic waves with phase shift are considered to be one of the key factors to cause small number of formed bubbles at the quenching condition.

## 4 Conclusion

In this study, we numerically investigated the influence of harmonic waves on rectified diffusion around an acoustic cavitation bubble. The Keller-Miksis equation and the advection diffusion equation for dissolved gas were solved simultaneously, and the bubble growth or dissolution rate in an ultrasonic cycle was calculated under the saturated gas condition. The findings in this study are summarized below.

• In the case with harmonic waves without any phase shift, the maximum bubble radius during an ultrasonic oscillation becomes small due to instantaneous pressurization, reducing the bubble growth due to rectified diffusion compared with the bubble growth without any harmonic wave.
• The bubble growth and dissolution due to rectified diffusion is mainly determined by the *area effect* under the condition where the dissolved oxygen is saturated.
• In the case with harmonic waves with phase shift, the bubble expansion is largely suppressed, reducing the bubble growth due to rectified diffusion particularly for *θ* = −π/2.
• The reason, why the power-induced quenching occurs under the condition where the ratio of harmonic waves is large and the number of formed bubbles is small, is considered to be caused by the decrease in the bubble growth due to smaller rectified diffusion, which is caused by the sound wave with harmonic waves and phase shift.

## CRediT authorship contribution statement

**Kanji D. Hattori:** Writing – original draft, Visualization, Validation, Software, Methodology, Investigation, Formal analysis, Data curation. **Takuya Yamamoto:** Writing – review & editing, Software, Project administration, Funding acquisition, Conceptualization.

## Declaration of competing interest

The authors declare the following financial interests/personal relationships which may be considered as potential competing interests: Takuya Yamamoto reports financial support was provided by Japan Science and Technology Agency. If there are other authors, they declare that they have no known competing financial interests or personal relationships that could have appeared to influence the work reported in this paper.

## References

[1] Yasui K. (2018).

[2] Leighton T.G. (1994).

[3] Xu H., Zeiger B.W., Suslick K.S. (2013). Sonochemical synthesis of nanomaterials. Chem. Soc. Rev..

[4] Eller A., Flynn H.G. (1965). Rectified diffusion during nonlinear pulsations of cavitation bubbles. J. Acoust. Soc. Am..

[5] Suslick K.S. (1990). Sonochemistry. Science.

[6] Lauterborn W., Kurz T. (2010). Physics of bubble oscillations. Rep. Prog. Phys..

[7] Matula T.J., Halla I.M., Cleveland R.O., Crum L.A., Moss W.C., Roy R.A. (1998). The acoustic emissions from single-bubble sonoluminescence. J. Acoust. Soc. Am..

[8] Philipp A., Lauterborn W. (1998). Cavitation erosion by single laser-produced bubbles. J. Fluid Mech..

[9] Bußmann A., Riahi F., Gökce B., Adami S., Barcikowski S., Adams N.A. (2023). Investigation of cavitation bubble dynamics near a solid wall by high-resolution numerical simulation. Phys. Fluids.

[10] Louisnard O. (2017). A viable method to predict acoustic streaming in presence of cavitation. Ultrason. Sonochem..

[11] Yamamoto T., Kubo K., Komarov S.V. (2021). Characterization of acoustic streaming in water and aluminum melt during ultrasonic irradiation. Ultrason. Sonochem..

[12] Canselier J.P., Delmas H., Wilhelm A.M., Abismaïl B. (2002). Ultrasound Emulsification—An Overview. J. Dispers. Sci. Technol..

[13] Udepurkar A.P., Clasen C., Kuhn S. (2023). Emulsification mechanism in an ultrasonic microreactor: Influence of surface roughness and ultrasound frequency. Ultrason. Sonochem..

[14] Raman K.A., Rosselló J.M., Ohl C.-D. (2022). Cavitation induced oil-in-water emulsification pathways using a single laser-induced bubble. Appl. Phys. Lett..

[15] Yamamoto T., Matsutaka R., Komarov S.V. (2021). High-speed imaging of ultrasonic emulsification using a water-gallium system. Ultrason. Sonochem..

[16] Zhang Y., Yuan S., Wang L. (2021). Investigation of capillary wave, cavitation and droplet diameter distribution during ultrasonic atomization. Exp. Therm Fluid Sci..

[17] Zhang Y., Yuan S., Gao Y. (2023). Spatial distribution and transient evolution of sub-droplet velocity and size in ultrasonic atomization. Exp. Therm Fluid Sci..

[18] Nguyen V.S., Rouxel D., Hadji R., Vincent B., Fort Y. (2011). Effect of ultrasonication and dispersion stability on the cluster size of alumina nanoscale particles in aqueous solutions. Ultrason. Sonochem..

[19] Sauter C., Emin M.A., Schuchmann H.P., Tavman S. (2008). Influence of hydrostatic pressure and sound amplitude on the ultrasound induced dispersion and de-agglomeration of nanoparticles. Ultrason. Sonochem..

[20] Priyadarshi A., Khavari M., Subroto T., Prentice P., Pericleous K., Eskin D., Durodola J., Tzanakis I. (2021). Mechanisms of ultrasonic de-agglomeration of oxides through in-situ high-speed observations and acoustic measurements. Ultrason. Sonochem..

[21] Yamanaka T., Hayashi Y., Takizawa H. (2023). Near-room temperature synthesis of Zn2+-doped γ-Ga2O3 nanoparticles via direct oxidation of Zn–Ga alloy by ultrasound. J. Ceram. Soc. Jpn..

[22] Maruthapandi M., Gupta A., Saravanan A., Jacobi G., Banin E., Luong J.H.T., Gedanken A. (2023). Ultrasonic-assisted synthesis of lignin-capped Cu(2)O nanocomposite with antibiofilm properties. Ultrason. Sonochem..

[23] Adewuyi Y.G. (2001). Sonochemistry: environmental science and engineering applications. Ind. Eng. Chem. Res..

[24] Okitsu K., Nanzai B., Kawasaki K., Takenaka N., Bandow H. (2009). Sonochemical decomposition of organic acids in aqueous solution: understanding of molecular behavior during cavitation by the analysis of a heterogeneous reaction kinetics model. Ultrason. Sonochem..

[25] Ilić N., Andalib A., Lippert T., Knoop O., Franke M., Bräutigam P., Drewes J.E., Hübner U. (2023). Ultrasonic degradation of GenX (HFPO-DA) – Performance comparison to PFOA and PFOS at high frequencies. Chem. Eng. J..

[26] Shende T., Andaluri G., Suri R. (2023). Chain-length dependent ultrasonic degradation of perfluoroalkyl substances. Chem. Eng. J. Adv..

[27] Hatanaka S.I., Yasui K., Tuziuti T., Kozuka T., Mitome H. (2001). Quenching mechanism of multibubble sonoluminescence at excessive sound pressure. Jpn. J. Appl. Phys..

[28] Hatanaka S.-I., Yasui K., Kozuka T., Tuziuti T., Mitome H. (2002). Influence of bubble clustering on multibubble sonoluminescence. Ultrasonics.

[29] Fattahi K., Boffito D.C., Robert E. (2024). Quantifying the chemical activity of cavitation bubbles in a cluster. Sci. Rep..

[30] Yasui K., Iida Y., Tuziuti T., Kozuka T., Towata A. (2008). Strongly interacting bubbles under an ultrasonic horn. Phys. Rev. E Stat. Nonlin. Soft Matter Phys..

[31] Asakura Y., Yasuda K. (2021). Frequency and power dependence of the sonochemical reaction. Ultrason. Sonochem..

[32] Tuziuti T., Yasui K., Sivakumar M., Iida Y., Miyoshi N. (2005). Correlation between acoustic cavitation noise and yield enhancement of sonochemical reaction by particle addition. Chem. A Eur. J..

[33] Tuziuti T., Yasui K., Kozuka T., Towata A. (2010). Influence of liquid-surface vibration on sonochemiluminescence intensity. J. Phys. Chem. A.

[34] Tuziuti T., Yasui K., Kozuka T., Towata A., Iida Y. (2007). Suppression of sonochemiluminescence reduction at high acoustic amplitudes by the addition of particles. J. Phys. Chem. A.

[35] Kojima Y., Asakura Y., Sugiyama G., Koda S. (2010). The effects of acoustic flow and mechanical flow on the sonochemical efficiency in a rectangular sonochemical reactor. Ultrason. Sonochem..

[36] Wood R.J., Lee J., Bussemaker M.J. (2019). Disparities between sonoluminescence, sonochemiluminescence and dosimetry with frequency variation under flow. Ultrason. Sonochem..

[37] Price G.J., Smith P.F. (1993). Ultrasonic degradation of polymer solution: 2. The effect of temperature, ultrasound intensity and dissolved gases on polystyrene in toluene. Polymer.

[38] Tuziuti T., Hatanaka S.I., Yasui K., Kozuka T., Mitome H. (2002). Influence of dissolved oxygen content on multibubble sonoluminescence with ambient-pressure reduction. Ultrasonics.

[39] Rooze J., Rebrov E.V., Schouten J.C., Keurentjes J.T. (2013). Dissolved gas and ultrasonic cavitation–a review. Ultrason. Sonochem..

[40] Lee J., Yasui K., Tuziuti T., Kozuka T., Towata A., Iida Y. (2008). Spatial distribution enhancement of sonoluminescence activity by altering sonication and solution conditions. J. Phys. Chem. B.

[41] Aoki R., Hattori K.D., Yamamoto T. (2025). Revisit to the mechanism of quenching: Power effects for sonochemical reactions. Ultrason. Sonochem..

[42] Aoki R., Yamamoto T. (2026). Interaction between number of formed bubbles, reaction rate and flow velocity of acoustic streaming in the initiation period of power-induced quenching. Ultrason. Sonochem..

[43] Hayashi R., Yamamoto T. (2026). Multiscale numerical simulation based on Caflisch model to interpret power-induced quenching for sonochemical reactions. Ultrason. Sonochem..

[44] Louisnard O., Gomez F. (2003). Growth by rectified diffusion of strongly acoustically forced gas bubbles in nearly saturated liquids. Phys. Rev. E Stat. Nonlin. Soft Matter Phys..

[45] Yoshizawa S., Takagi S., Matsumoto Y. (2009). Growth of an oscillating microbubble in an ultrasound field (Enhancement of rectified diffusion with a dual-frequency excitation method). Trans. JSME (b).

[46] Zhang Y., Zhang Y. (2019). Rectified diffusion of gas bubbles in molten metal during ultrasonic degassing. Symmetry.

[47] Hilgenfeldt S., Lohse D., Brenner M.P. (1996). Phase diagrams for sonoluminescing bubbles. Phys. Fluids.

[48] Smith W.R., Wang Q.X. (2023). The role of surface tension on the growth of bubbles by rectified diffusion. Ultrason. Sonochem..

[49] Smith W.R., Wang Q. (2022). A theoretical model for the growth of spherical bubbles by rectified diffusion. J. Fluid Mech..

[50] Keller J., Miksis M. (1980). Bubble oscillations of large amplitude. J. Acoust. Soc. Am..

[51] Hattori K.D., Yamamoto T. (2025). Stability analysis of the effect of harmonic waves on the shape stability of acoustic cavitation bubbles. Ultrason. Sonochem..

[52] Yasui K. (2001). Effect of liquid temperature on sonoluminescence. Phys. Rev. E Stat. Nonlin. Soft Matter Phys..

[53] Yasui K. (1996). Variation of liquid temperature at bubble wall near the sonoluminescence threshold. J. Phys. Soc. Jpn..

[54] Yamamoto T. (2024). Linear stability analysis for bubble shape of acoustic cavitation with different ultrasonic frequencies. Phys. Fluids.

[55] Yamamoto T. (2024). Bubble shape instability of acoustic cavitation in molten metal used in ultrasonic casting. Ultrason. Sonochem..

